# Structure of *Aquifex aeolicus* lumazine synthase by cryo-electron microscopy to 1.42 Å resolution

**DOI:** 10.1107/S2052252524005530

**Published:** 2024-07-04

**Authors:** Christos G. Savva, Mohamed A. Sobhy, Alfredo De Biasio, Samir M. Hamdan

**Affiliations:** a Biological and Environmental Science and Engineering King Abdullah University of Science and Technology 4700 KAUST Thuwal23955 Saudi Arabia; Boston University School of Medicine, USA

**Keywords:** cryo-EM, atomic resolution, *Aquifex aeolicus*, lumazine synthase, single-particle analysis

## Abstract

Single-particle cryo-electron microscopy (cryo-EM) has become an essential structural determination technique with recent hardware developments making it possible to reach atomic resolution, at which individual atoms, including hydrogen atoms, can be resolved. In this study, we used the enzyme involved in the penultimate step of riboflavin biosynthesis as a test specimen to benchmark a recently installed microscope and determine if other protein complexes could reach a resolution of 1.5 Å or better, which so far has only been achieved for the iron carrier ferritin. Using state-of-the-art microscope and detector hardware as well as the latest software techniques to overcome microscope and sample limitations, a 1.42 Å map of *Aquifex aeolicus* lumazine synthase (AaLS) was obtained from a 48 h microscope session. In addition to water molecules and ligands involved in the function of AaLS, we can observe positive density for ∼50% of the hydrogen atoms. A small improvement in the resolution was achieved by Ewald sphere correction which was expected to limit the resolution to ∼1.5 Å for a molecule of this diameter. Our study confirms that other protein complexes can be solved to near-atomic resolution. Future improvements in specimen preparation and protein complex stabilization may allow more flexible macromolecules to reach this level of resolution and should become a priority of study in the field.

## Abstract 2

A near-atomic resolution map was obtained for lumazine synthase while benchmarking a new microscope. At this resolution, waters, ligands and hydrogens were visible. A detailed outline of the methods used is presented that can employed for any single-particle cryo-EM experiment.

## 1. Introduction

Single-particle cryo-EM has advanced at a fast pace over the last 10 years beginning with the first high-resolution structures in 2013 using direct detection devices (Bai *et al.*, 2013 ▸; Li *et al.*, 2013 ▸; Liao *et al.*, 2013 ▸) referred to as the ‘resolution revolution’ (Kühlbrandt, 2014 ▸). The exponential growth of the field is highlighted by the number of deposited structures in the Electron Microscopy Database (EMDB), which surpassed 30 000 cumulative maps in 2023 and with almost 25% of these being deposited during that same year (source: EMDB statistics; March 2024). This highlights the adoption of cryo-EM as a mainstream structural biology determination approach by existing and newly established structural biology groups around the world. Furthermore, the rapid development of user-friendly software for high-speed data collection and the creation of national or regional facilities worldwide have made the process of collecting high-quality data more tangible than ever before. Recently, the potential of a low-cost screening/data collection microscope was demonstrated which will enable the dissemination of the technique to more researchers (McMullan *et al.*, 2023 ▸).

Although significant hurdles still exist in obtaining suitable samples for cryo-EM with few cases being straight forward and requiring an optimization process by trial and error, the potential of atomic-resolution single-particle analysis (SPA; *i.e.* close to 1.2 Å) was demonstrated on the gold-standard apoferritin by several groups beginning in 2020 (Nakane *et al.*, 2020 ▸; Yip *et al.*, 2020 ▸). The recombinant version of either mouse or human ferritin can routinely reach resolutions higher than 2 Å on field emission gun (FEG) microscopes, whereas use of a highly coherent cold field emission gun (cFEG) or a monochromator and spherical aberration corrector greatly increase the signal attainable near the 1 Å resolution range (Nakane *et al.*, 2020 ▸). The use of these latest hardware allowed the structures of apoferritin to be determined to 1.22 and 1.25 Å, respectively, while in 2023 a 1.19 Å map was also reported (Maki-Yonekura *et al.*, 2023 ▸). This leap in resolution resulted in maps in which individual atoms could be placed unambiguously and the visualization of hydrogen atom positions by calculating difference maps between the experimental density and atomic coordinates is possible (Yamashita *et al.*, 2021 ▸). More recently, other (non-test specimen) SPA-derived structures have also approached near-atomic resolution including the 1.55 Å structure of a prokaryotic ribosome (Fromm *et al.*, 2023 ▸) and the 1.52 Å structure of the *M. smegmatis* Huc complex (Grinter *et al.*, 2023 ▸).

The Imaging and Characterization Core Lab at the King Abdullah University of Science and Technology (KAUST) acquired a Thermo–Fisher Scientific (TFS) Krios G4 in the summer of 2022 to enable high-throughput cryo-EM for the users in the University and wider region. During commissioning of the microscope, a 2.0 Å map of mouse apoferritin was obtained using aberration free image shift (AFIS). The Krios G4 equipped with a cFEG source, Selectris-X post-column energy filter and a Falcon 4i detector is almost identical to the setup which resulted in the 1.22 Å apoferritin structure and therefore we wanted to repeat a benchmark to identify any potential issues with this microscope and explore its capabilities.

A previous study conducted locally on an older Krios G1 upgraded with a Gatan imaging filter and a Gatan K2 detector explored the suitability of two high-symmetry protein complexes as benchmark candidates (Sobhy *et al.*, 2022 ▸). Of these, AaLS from the hyper-thermophilic bacterium *Aquifex aeolicus* forms a 1 MDa spherical capsid of 60 identical subunits with icosahedral symmetry (Zhang *et al.*, 2001 ▸). Using this older hardware, a reconstruction of 2.3 Å was achieved which was very close to Nyquist for the pixel size used. Therefore, we opted to use AaLS as a test specimen to evaluate the Krios G4.

## 2. Methods

### 2.1. Protein purification

The amino-acid sequence of the lumazine synthase gene from *Aquifex aeolicus* (AaLS) (Uniprot O66529) fused to a Streptag at the C-terminal was codon-optimized for expression in *Escherichia coli*. The gene was ordered from Integrated DNA Technologies (IDT) and cloned using Gibson assembly. The sequence of the gene in the transformed plasmid was then verified by Sanger sequencing. The transformed BL21 (DE3) cells were grown in LB media at 37°C until reaching an optical density at 600 nm wavelength (OD_600_) of 0.8. Protein expression was induced using 0.2 m*M* IPTG concentration in the culture. The expression was done at 16°C for a duration of 17 h. The spun-down cell pellet was resuspended in lysis buffer [100 m*M* potassium phosphate (KP_i_) buffer pH 8, 1%(*v*/*v*) Tween 20, 50 µ*M* EDTA and protease inhibitor]. The cells were disrupted by ultrasonication and the supernatant was heated at 75°C for 45 min then spun down and filtered through a 45 µm filter to remove precipitated heat-labile proteins. The filtrate was passed through a StrepTrap HP 5 ml column (GE Healthcare) using 100 m*M* Tris–HCl pH 8.0 and 150 m*M* NaCl (Buffer A). AaLS was eluted by Buffer B (Buffer A + 2.5 m*M* d-desthio­biotin). The fractions containing the protein were collected and concentrated using 100 kDa cutoff concentrator and loaded onto a Superdex 200 10/300GL column (GE Healthcare) using 20 m*M* Tris–HCl pH 7.5 and 150 m*M* NaCl. The fractions containing the eluted protein were curated and concentrated using 100 kDa cutoff concentrator then flash-frozen into liquid nitro­gen and stored at −80°C.

### 2.2. Electron microscopy

Frozen aliquots of AaLS were thawed on ice and diluted to 2.75 mg ml^−1^ in 20 m*M* Tris pH 7.5, 150 m*M* NaCl and 1 m*M* DTT. UltrAuFoil (Quantifoil GmbH) R1.2/1.3 Au 300 mesh grids were first washed in acetone for 30 s followed by iso­propyl alcohol for 10 s and left to dry. Cleaned grids were glow-discharged for 30 s at 30 mA in air using a PELCO easiGlow unit. A TFS Vitrobot MKIV was then used to plunge-freeze grids in liquid ethane (3 µl sample, 2 s blot time, blot force −5, 100% humidity). Grids were loaded onto a TFS Krios G4 located at the Imaging and Characterization Core Lab at KAUST equipped with a cFEG, fringe-free illumination (FFI) and a Falcon 4i direct detection device mounted at the end of a Selectris-X post-column energy filter.

Prior to data collection, the microscope was setup as follows. A new gain reference was acquired at a flux of 5 e^−^pixel^−1^ s^−1^. This was followed by tuning of the energy filter at the data collection magnification. Using the *Sherpa* software (TFS), tuning was performed to correct for non-isochromaticity, and geometrical and chromatic distortions as per the manufacturer’s application notes. The cFEG was then flashed to ensure maximum emission prior to setup of data collection and to measure the maximum fluence the sample would receive during collection. A grid with carbon was then loaded and used for all alignments. The eucentric height was set over an area with carbon and the optics for data collection (magnification, spot size, illumination area) were applied to the microscope. True focus was set on the objective lens followed by a defocus of approximately −1.0 µm. *AutoCTF* (TFS) was used to correct for objective lens astigmatism followed by automatic beam-tilt correction (coma). Finally, the defocus was set to approximately −0.5 µm and to correct for residual objective lens astigmatism.

In total, 12 657 movies were recorded over a 48 h period. A nominal magnification of 270 000× resulted in a calibrated physical pixel size of 0.4553 Å (calibrated as described below). The 50 µm second condenser aperture was used and an objective aperture was omitted entirely so as not to limit the attainable resolution (100 µm aperture high-frequency cut-off ∼1.4 Å). A flux of 3.1 e^−^pixel^−1^ s^−1^ (as measured on the detector over vacuum) resulted in a total fluence of ∼46 e^−^ Å^−2^ on the specimen over a 3 s exposure. A total of 918 frames were saved in EER format. All grid screening and data collection were carried out using EPU (version 3.5.1, Thermo–Fisher Scientific) and using stage shift rather than AFIS to minimize the effect of beam-tilt. A 5 s stage settling time was used between stage shifts. A parallel beam diameter of 350 nm, confirmed over gold foil in diffraction mode, allowed the exposure of nine areas within a hole using beam-image shift. A nominal defocus range −1.2 to −0.4 µm in 0.2 µm intervals was applied over the dataset and the energy filter slit width was set to 10 eV without automatic re-centering of the zero-loss energy peak.

### 2.3. Data processing

Prior to data collection, five drift-correct exposures were recorded over the gold foil using the same illumination conditions as data collection. The images were then imported into *magCalEM* (version 13.0; Dickerson *et al.*, 2024 ▸). The program provides a calculated pixel size, taking into account any potential magnification anisotropy of the microscope projection system. At the time of acquisition, the calibrated pixel size was measured to be 0.4553 Å, 1.2% larger than the service calibrated pixel of 0.45 Å.

*RELION* (version 5.0b; Scheres, 2012 ▸) was used for all processing steps described below. EER format movies were gain- and motion-corrected using the *RELION* implementation with an EER fractionation group size of 18 raw frames resulting in 51 fractions with a fluence of 0.88 e^−^ Å^−2^ per fraction. Contrast transfer function (CTF) estimation was carried out using *CTFFIND4* (Rohou & Grigorieff, 2015 ▸) followed by curation of micrographs based on resolution (better than 5 Å), defocus (−0.2 to −1.7 µm), relative figure of merit and finally manual removal of images with significant crystalline ice present. Log-based autopicking was used to pick and generate a set of 2D classes for reference-based picking. In total, 698 240 particles were extracted and downscaled 4×. Following on from 2D classification, particles were extracted at the full pixel size in a 700 pixel box and reached a 3D auto-refine resolution of 1.97 Å using icosahedral (I) symmetry. All refinement steps were performed using a soft mask on the reference and calculating solvent-flattened FSCs. A first round of CTF refinement was carried out in the following order. First, the refined particles were used to estimate beam tilt (Zivanov *et al.*, 2018 ▸), threefold astigmatism and fourth-order aberrations (Zivanov *et al.*, 2020 ▸). This was followed by the estimation of magnification anisotropy (Zivanov *et al.*, 2020 ▸) and finally per-particle defocus and per-micrograph astigmatism (Zivanov *et al.*, 2018 ▸). This led to a map at 1.75 Å resolution. Bayesian polishing (Zivanov *et al.*, 2019 ▸) improved the resolution slightly to 1.72 Å while a second round of CTF refinement (same procedure as before) resulted in a significant increase to 1.46 Å owing most likely to the higher-resolution reference map. As a final step to sort out particle heterogeneity, 3D classification without alignment, and using a regularization parameter of *t* = 10 and two classes resulted in a subset of ∼80% of particles of higher resolution that were subjected to a final round of the same CTF refinement routine leading to a final map at 1.43 Å. Note that the final beam tilt and magnification anisotropy estimated values were very similar to the second round of CTF refinement but different to the first round (∼50% difference for beam tilt and ∼10% difference for anisotropy). Using the refinement *run_data.star* to reconstruct the particles while taking into consideration the Ewald Sphere (Russo & Henderson, 2018 ▸; Zivanov *et al.*, 2018 ▸) resulted in a final map at 1.42 Å resolution. All post-processing steps took into account the calibrated pixel size and the modulation transfer function (MTF) of the detector. The estimated beam tilt for this dataset was *X* = −0.02206 mrad and *Y* = −0.02670 mrad and the estimated magnification anisotropy was 1.75%. Subsets of particles were used for the Rosenthal *B* factor estimation (Rosenthal & Henderson, 2003 ▸) from an initial random selection of 240 000 from the final 470 878 particle set. Each subset was refined against a 30 Å filtered reference and post-processed as above.

### 2.4. Model building and refinement

*Servalcat*/*Refmac5* (Yamashita *et al.*, 2021 ▸; Murshudov *et al.*, 2011 ▸), as implemented in the *CCPEM* suite (Burnley *et al.*, 2017 ▸), was used for model refinement in combination with *Coot* (Emsley *et al.*, 2010 ▸) for manual model building and inspection. The AaLS 1.60 Å crystal structure monomer (PDB entry 1hqk; Zhang *et al.*, 2001 ▸) was stripped of water molecules and docked in UCSF *Chimera* (Pettersen *et al.*, 2004 ▸) to one asymmetric unit of the map. Unsharpened and unweighted half maps from the Ewald sphere-corrected reconstruction and this model were used for ten cycles of refinement with autosymmetry set to the Global and strict icosahedral (I) point group symmetry. This allowed us to work with one monomer while using a symmetry-expanded model for refinement. Waters in the sharpened map were identified in *Coot* within a distance of 2.0–3.2 Å of the protein atoms and were added to this model. This hydrated model was used for another round of *Servalcat* refinement and the resulting difference (*F*_o_ − *F*_c_) map calculated between the map and input model were masked around one asymmetric unit using *RELIONMask Create* and *Map Process* in *CCPEM*. *PEAKMAX* from the *CCP4* suite (Agirre *et al.*, 2023 ▸) was used to identify peaks above 2.0 s in the *F*_o_ − *F*_c_ map followed by automatic selection of peaks in *WATPEAK* (*CCP4*) of less than 0.5 Å from atoms in a model with added hydrogens in all possible positions. Approximately 46% of all possible hydrogens could be accounted for in the difference map (552 out of 1190).

## 3. Results

**Figure 1:**
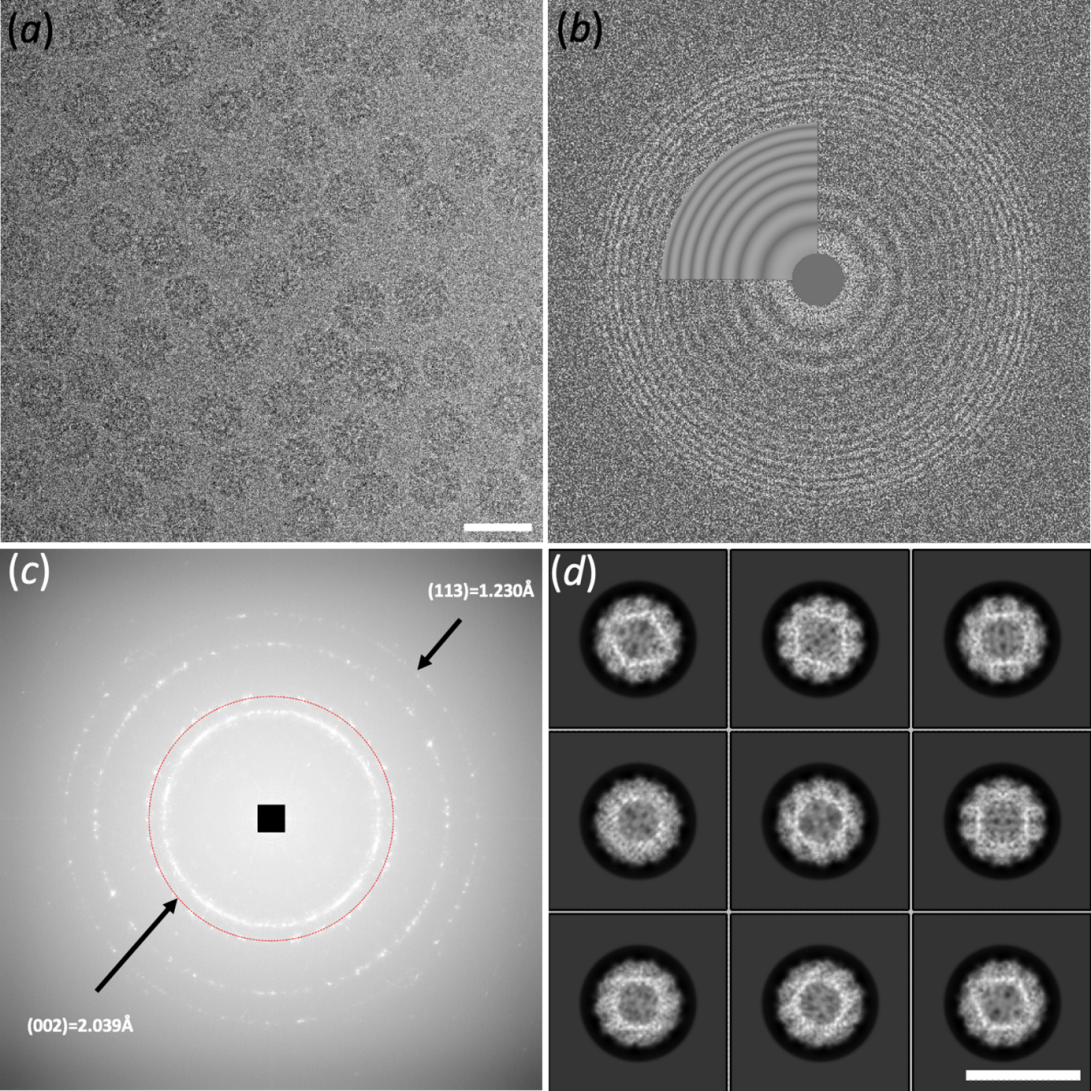
Cryo-EM of AaLS. (*a*) Representative electron micrograph of AaLS particles taken at an approximate defocus of −1.0 µm and an approximate total fluence of 46 e^−^ Å^−2^. (*b*) Corresponding CTF parameter estimation power spectrum. (*c*) Summed power spectrum of five micrographs of UltrAuFoil support film indicating the (002) diffraction ring (red circle) used for pixel calibration. The (113) ring is also visible, indicating frequencies to 1.23 Å using conditions identical to data collection. (*d*) Class averages of 4× binned AaLS particles. Scale bars correspond to 200 Å.

Using the same protein preparation of AaLS used for the 2.3 Å Krios G1-derived map, we prepared specimens using UltrAuFoil grids to minimize beam-induced particle movement (Russo & Passmore, 2014 ▸). Careful alignment of the microscope optics to minimize objective lens astigmatism and axial coma were performed on grids coated with holey carbon immediately prior to the data collection setup. The choice of suitable squares is very important when trying to obtain the best performance from the microscope and detector as increased ice thickness can be detrimental to high-resolution structural determination. Several studies have been conducted to optimize square and hole selection using different aspects that are affected by sample thickness such as loss of electrons by either inelastic scattering or high-angle scattering through the objective aperture. These measurements, combined with experimental determination of ice thickness, can be used to calibrate on-the-fly ice thickness determination parameters (Rice *et al.*, 2018 ▸; Rheinberger *et al.*, 2021 ▸). Another report used Plasmon range energy-loss electrons to aid hole selection especially on gold foils where the increased thickness of the foil makes determining the ice thickness more challenging (Hagen, 2022 ▸). In practice, the ice thickness parameter must be taken into consideration alongside particle distribution and stability. In *TFS EPU* (version 3.5), a per-hole histogram function which relays the grey-level distribution in individual holes allows one to set the minimum and maximum grey-level range from such holes to the entire dataset. The reported fluence in EPU can then be used to find the thinnest possible ice (by comparing to vacuum) that gives a good distribution of particles. We have routinely used this approach when using UltrAuFoil grids to aid in hole selection and this approach was implemented for this dataset. From the resulting micrographs, 95% displayed CTF estimated frequencies better than 5 Å and reaching ∼2.5 Å in the best cases [Figs. 1 ▸(*a*) and 1 ▸(*b*)].

The calibrated pixel size is an important factor when reporting the resolution of cryo-EM data and especially when trying to obtain high resolution. In addition to causing fitting errors of the contrast transfer function (CTF) at higher frequencies (Dickerson *et al.*, 2024 ▸; Danev *et al.*, 2021 ▸), the pixel size determines the reported resolution. Even if fourth-order aberration estimation is carried out during processing, which can mitigate the effect of an incorrect pixel size (Zivanov *et al.*, 2020 ▸), the input of the calibrated pixel size during post-processing is important for the correct scaling of maps to be used in model refinement and accurate reporting of resolution. As reported by others (Danev *et al.*, 2021 ▸; Dickerson *et al.*, 2024 ▸), the nominal calibrated pixel size which is determined by service engineers can be off by several percentiles. In addition, pixel calibration using grating replicas of varying ratios of metals such as gold/palladium affects the position of the diffraction rings depending on this ratio and care should be taken to use pure metals for this purpose (Danev *et al.*, 2021 ▸). Finally, the projection systems of microscopes are not tunable by the user and varying amounts of anisotropic magnification can be present from the factory (Zivanov *et al.*, 2020 ▸; Grant & Grigorieff, 2015 ▸). The recently reported *magCalEM* software package has been developed specifically for calibrating the correct pixel while taking into account the effect of anisotropic magnification (Dickerson *et al.*, 2024 ▸). We used this software to obtain the correct pixel size which in our case was 1.2% larger than the service determined pixel size [Fig. 1 ▸(*c*)]. The calculated power spectra of the UltrAuFoil support film indicated frequencies at least to 1.23 Å [Fig. 1 ▸(*c*)] under identical optics and fluence used for data collection.

We opted to use stage shift for this experiment to reduce the effect of coma on the datasets as reported by Nakane *et al.* (2020 ▸). As the G4 provides FFI, a square pattern of nine exposures could be accommodated in each hole using a 350 nm parallel beam with no contact between the beam and foil and no overlap of the illuminated areas. The flux on the Falcon4i detector was set to ∼3 e^−^ pixel^−1^ s^−1^ or approximately one electron per hundred pixels per frame to minimize coincidence loss (Greg McMullan, personal communication; TFS Falcon4i Applications Notes). Over the 48 h session, 12 657 movies were recorded in EER format (Guo *et al.*, 2020 ▸). Data collection parameters are summarized in Table 1 ▸.

**Figure 2:**
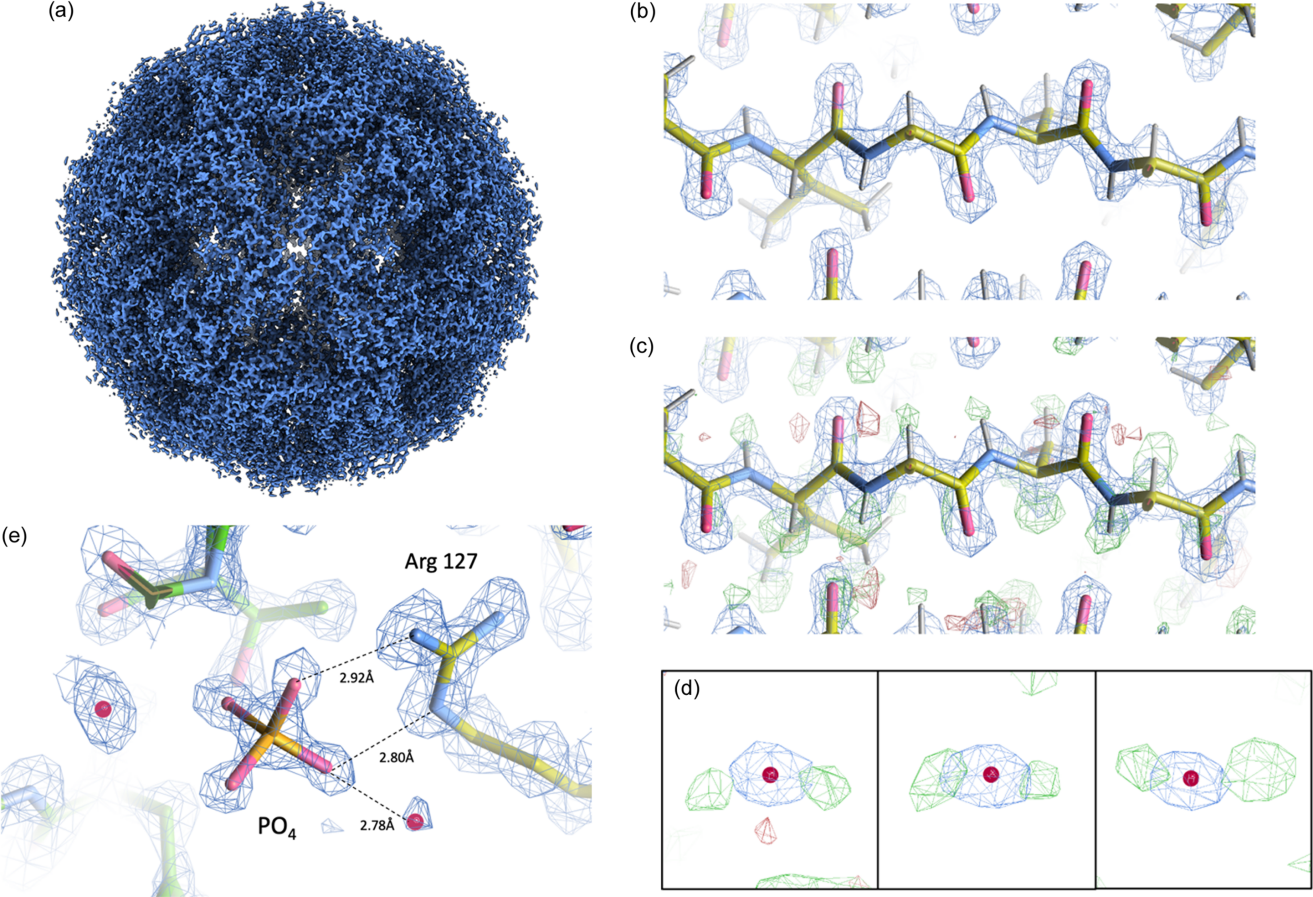
Map and model of AaLS. (*a*) Sharpened map of AaLS at 1.42 Å resolution. (*b*) Density at 4.0σ of a masked-normalized *F*_o_ map output from *Servalcat*. (*c*) Overlay of the calculated *F*_o_ − *F*_c_ difference map at 4.0σ, (*b*) indicating positive density (green) and in some areas negative density (red). Hydrogen atoms were built into the model if the corresponding positive peaks were within 0.5 Å of the putative hydrogen atoms. (*d*) Examples of water molecules identified with accompanying hydrogen atoms. (*e*) Arginine 127 interacting with a phosphate ion which is a bi-product of lumazine synthesis. Potential hydrogen-bonding distances are indicated by dashed lines.

The final 3D reconstruction of AaLS consisted of 470 878 particles. This 1.43 Å map was slightly improved by Ewald sphere correction to 1.42 Å [Figs. 2 ▸(*a*), and S1(*a*) and S1(*b*) of the supporting information] (Zivanov *et al.*, 2018 ▸; Russo & Henderson, 2018 ▸). AaLS has a diameter of 160 Å and the effect of the Ewald Sphere is expected to start affecting frequencies higher than 1.5 Å (DeRosier, 2000 ▸). In this case, it seems that the effect was not as prominent but it is possible that further improvement could be limited by the flexibility of the protein itself or the signal in the data. The Rosenthal *B* factor (Rosenthal & Henderson, 2003 ▸) was calculated to be 49 Å^2^ using subsets of particles [Fig. S1(*d*)]. The smallest subset consisting of 936 particles resulted in a 2.01 Å map.

The half maps from Ewald sphere correction were subsequently used for atomic model refinement using the 1.60 Å crystal structure of AaLS as a starting model. Water molecules were identified in the sharpened map and of these, 43 were within 0.2 Å of the water molecules modelled in the crystal structure (PDB entry 1hqk). A mask-normalized *F*_o_ − *F*_c_-generated difference map was then used to identify peaks within 0.5 Å of hydrogen atoms in a reduced model, followed by manual curation which resulted in 552 hydrogens. Thus, we were able to identify ∼46% of all putative protein hydrogen atoms from the map of AaLS at this resolution [Figs. 2 ▸(*b*) and 2 ▸(*c*)]. This number is consistent with previous studies using apoferritin maps determined at different resolutions. About 70% of hydrogen atoms could be located in maps at 1.19 and 1.25 Å resolution (Maki-Yonekura *et al.*, 2023 ▸; Yamashita *et al.*, 2021 ▸) compared with only ∼17% at 1.84 Å resolution (Yamashita *et al.*, 2021 ▸). Several of the water molecules identified displayed hydrogen densities adjacent to the central oxygen atom [Fig. 2 ▸(*d*)]. Finally, we observed the density for a tetrahedral-shaped ligand. This was built as a phosphate ion and is consistent as a bi-product of lumazine synthesis and in proximity to Arginine 127, a highly conserved residue in this family of proteins [Fig. 2 ▸(*e*)] (Zhang *et al.*, 2001 ▸). At pH 7.5 the phosphate ion is expected to be mostly in the HPO_4_^−2^ proton­ation state, though we did not observe positive density for any hydrogens. It is unknown whether the phosphate is a result of enzymatic catalysis or simply carried over from phosphate present in the lysis buffer. Fourier shell correlation between the final map and model as well as cross-validation FSCs indicated no overfitting during refinement [Fig. S1(*c*)] (Brown *et al.*, 2015 ▸). Model refinement statistics are shown in Table 2 ▸.

## 4. Discussion

In this study, we set out to benchmark a recently installed, state-of-the-art microscope and to identify any potential issues. Previously, and using older hardware and data collection methodology, a map of AaLS obtained by Sobhy *et al.* (2022 ▸) reached a moderately high resolution of 2.3 Å, which was very close to Nyquist. The estimated Rosenthal *B* factor for that dataset was 110 Å^2^ [Fig. S1(*d*)]. The use of AaLS as a test sample allowed us to further examine its suitability as a viable benchmark specimen for higher-resolution structural determination. The structure we obtained from a 48 h session at 1.42 Å is to date the only sub-1.5 Å structure of a complex other than apoferritin. Though apoferritin has long been used as an ideal benchmark sample due to its stability, homogeneity and symmetry, there is no reason why other complexes with the same characteristics should not reach higher resolution if not limited by size. The calculated Rosenthal *B* factor from this dataset (49 Å^2^) [Fig. S1(*d*)] indicates that, though the data is of high quality, an impractical number of particles would be required to achieve significantly higher resolution. For example, to improve the resolution by 0.2 Å would require ∼1.6× more particles whereas reaching 1.2 Å would require an unrealistic 71 × 10^6^ particles. It is possible that other benchmarks of AaLS could have improved *B* factors that would match or surpass those obtained for apoferritin.

Though we did not use AFIS for this experiment, a collection speed of >300 movies per hour was achieved owing to the shorter exposure times at high magnification and the ability to use minimal beam-image shift within a hole with a very small, yet parallel beam. We anticipate that a properly calibrated AFIS routine and separation into optics groups could yield similar results. We identified a rather high-magnification anisotropy (∼1.7–1.8%) at the magnification used for this dataset (as well as a similar dataset 6 months prior). Though this can be corrected for *in silico*, the ultimate performance of the microscope could be compromised and requires further investigation. This highlights why benchmarking a newly installed microscope is important to any facility as no two identical hardware configurations behave exactly the same. Factory assembly, commissioning and environmental differences can affect microscope performance which can affect results at even intermediate resolution, as reported previously (Grant & Grigorieff, 2015 ▸).

In summary, we have demonstrated that SPA at near-atomic resolution is achievable for macromolecules other than apoferritin and can be obtained from user-friendly hardware configurations located at University Core facilities. Momentum should be directed towards sample optimization and novel vitrification approaches to obtain high-quality samples.

## 5. Data availability

Cryo-EM half maps have been deposited in the Electron Microscopy Data Bank (EMDB) under accession No. EMD-39478. The model has been deposited in the Protein Data Bank as PDB entry 8yt4.

## Supplementary Material

Supporting figure. DOI: 10.1107/S2052252524005530/eh5018sup1.pdf

PDB reference: *Aquifex aeolicus* lumazine synthase to 1.42 Å resolution, 8yt4

EMDB reference: *Aquifex aeolicus* lumazine synthase, EMD-39478

## Figures and Tables

**Table 1:** Cryo-EM data collection parameters

Microscope	TFS Titan Krios G4
Detector/energy filter	TFS Falcon 4i/Selectris-X
Magnification (nominal)	270000×
Energy filter slit width (eV)	10
Flux over vacuum (e^−^ pixel^−1^ s^−1^)	3.1
Total fluence per movie (e^−^ Å^−2^)	46
Exposure time (s)	3
EER frames per movie	918
Defocus range (µm) (nominal)	0.4–1.2
Calibrated pixel size (Å)	0.4553
Total movies collected	12657
Average collection speed per hour	315

**Table 2:** Model refinement statistics

Model resolution (Å)	1.4
Model resolution range (Å)	1.4–180.30
Mean overall *B* value (Å^2^)	20.046
Model composition	
Non-hydrogen atoms	1221
Hydrogen atoms	552
Protein residues	153
Ligands	1
R.m.s. deviations	
Bond lengths (Å)	0.006
Bond angles (°)	1.456
Validation	
Clashscore (No./1000 atoms)	1.7 (99th percentile)
Poor rotamers (%)	0.85
*MolProbity* score	0.92 (100th percentile)
Ramachandran plot	
Favored (%)	98
Allowed (%)	2
Outliers (%)	0
Cβ deviations (>0.25 Å)	0
Cis-prolines	0
